# Editorial: Alternatives to combat bacterial infections, volume II

**DOI:** 10.3389/fmicb.2023.1223241

**Published:** 2023-06-09

**Authors:** Luis Esaú López-Jácome, Mariano Martínez-Vázquez, Rodolfo García-Contreras

**Affiliations:** ^1^División de Infectología, Instituto Nacional de Rehabilitación Luis Guillermo Ibarra Ibarra, Mexico City, Mexico; ^2^Departamento de Productos Naturales, Instituto de Química, Universidad Nacional Autónoma de México, Mexico City, Mexico; ^3^Departamento de Microbiología y Parasitología, Facultad de Medicina, Universidad Nacional Autónoma de México, Mexico City, Mexico

**Keywords:** nanoparticles, photoinactivation, adjuvants, vaccines, infection diagnosis

## Introduction

The high prevalence of antibiotic resistance in pathogenic bacteria and its continuous increase are a threat for global health; hence, the development of alternatives to combat them is mandatory. In the first volume of this series, 30 innovative approaches to dealing with antibiotic resistant bacteria were presented (García-Contreras et al., 2022), while this second volume presents six new contributions.

## Nanoparticles

Metallic nanoparticles have bacteriostatic, bactericidal, and antibiofilm activities; however, for achieving biofilm eradication, their useful concentrations are high. Alves-Barroco et al. tested an alloy of gold–silver nanoparticles at growth sub-inhibitory concentrations against biofilms of *Streptococcus dysgalactiae, Pseudomonas aeruginosa*, and *Staphylococcus aureus* in combination with visible light irradiation and ciprofloxacin. Light irradiation heats the nanoparticles that then transfer heat to the biofilm matrix, disturbing it and increasing antibiotic penetration. The authors found that for some strains the treatment eliminated biofilms, and they suggest that biofilms matrixes with higher concentrations of extracellular DNA would be more susceptible.

There are several factors determining the effect of nanoparticles, such as size and shape (Albanese et al., 2012). Another important factor is the kind of metal used to synthesize the nanoparticles. Predominantly, silver was used, but other metals such as zinc and nickel have also been used (Vimbela et al., 2017; Mitra et al., 2020; Campo-Beleño et al., 2022; Zarenezhad et al., 2022). Dove et al. focused on the effect of nanoparticles and how they can enhance their activity in combination with antibiotics. The nanoparticles used were spherical with an average size of 2.53 nm. They tested several kinds of antibiotics, and the most effective were aminoglycosides. Moreover, they explored the possible adverse effects of the nanoparticles by assessing their toxicity in cellular lines and in animal models.

## Photoinactivation

In addition to the chemical antibacterial strategies, physical and biological methods were tested. Among the physical strategies, photo-inactivation seemed very promising. Gierke and Hessling studied the effect of light at wavelengths of around 222–254 nm on microorganisms. Ultraviolet-C radiation is used in laboratory components such as biosecurity cabinets and in hospitals to irradiate operating and patients' rooms. The use of bacterial pathogens is risky and difficult for some laboratories; hence, the authors proposed the use of non-pathogenic bacteria closely related with members of the ESKAPE group. They controlled variables such as microbial charge, time of light exposition, and wavelength. The use of surrogate microorganisms to perform and validate experiments increases the feasibility in terms of safety and efficacy to evaluate new antibacterial strategies. If the results are promising, the experiments can later be performed on the pathogens to validate the results.

## Adjuvants

Vaccination is perhaps the most effective way to minimize the effects of infectious diseases and to decrease their transmission. Tuberculosis is responsible for more than a million deaths per year. Currently the Bacille Calmette–Guérin (BCG) vaccine is used; however, the protection provided by this vaccine is limited since it is unable to prevent primary infections and the reactivation of latent pulmonary infections. Hence, some new vaccines are being developed, and among them, the ID93+ GLA-SE protein adjuvant vaccine that is currently being tested is in phase 2 clinical trials. ID93 is a recombinant protein composed of elements of four immunogenic *Mycobacterium tuberculosis* proteins. Baldwin et al. tested new adjuvant formulations with ID93 consisting of liposomal preparations in combination with QS-21 and a plant extract derived from the bark *Quillaja saponaria*, and they tested their effect to protect mice from a pulmonary *M. tuberculosis* infection. The formulations with anionic or neutral liposomes with QS-21 were able to remarkably increase mice survival and improve the immune response, encouraging further optimization and eventual utilization of this vaccine in humans.

## Antibiotic enhancers

Fatty acids (FAs) have antibacterial activities at high concentrations since they disrupt membranes causing leakage of intracellular metabolites. Various synthetic and natural compounds have been recently proposed to be used as antibiotic enhancers against *S. aureus* strains.

Park et al. found that FAs, particularly myristoleic acid, enhance the bactericidal activities of aminoglycoside antibiotics against *S. aureus* and that combinatory treatment of myristoleic acid and these antibiotics significantly decreases its biofilm formation. Moreover, several FAs such as oleic acid, cis-11-eicosenoic acid, and some long-chain omega-3 FAs have antibiofilm activity and reduce hemolytic activity (Kim et al., 2018). Furthermore, myristoleic acid inhibits the formation of *C. acnes* and *S. aureus* mixed biofilms, while lauric and myristic acids inhibit polymicrobial biofilms (Kim et al., 2021).

## Diagnosis

Early identification of bacterial cross-transmission events is crucial to prevent or minimize the magnitude of infection outbreaks, particularly in hospitals. The gold standard method for outbreak confirmation is whole-genome sequencing, which despite being very precise is relatively time consuming and costly and requires bioinformaticians to analyze the data. Hence, the implementation of a rapid and precise method for outbreak detections would allow a rapid identification of such events. Wang-Wang et al. demonstrated the potential of Fourier transform infrared spectroscopy (FTIR) for the quick identification of *Klebsiella pneumoniae* strains producing extended-spectrum β-lactamase. FTIR generates an infrared absorption spectrum of bacterial polysaccharides, allowing bacterial discrimination and typing. The FTIR technique could be used in conjunction with genome sequencing as a very efficient tool in the detection and identification of the bacterial outbreak.

## Conclusion

This editorial presents six excellent approaches covering diverse new strategies to combat bacterial infections. From the utilization of nanoparticles and their synergistic effects with light irradiation and antibiotics to the improvement of an experimental vaccine against tuberculosis, it highlights some of the groundbreaking contributions in the field of bacterial infections and drug resistance. The enhancement of antibiotic effects by fatty acids is an innovative method to supplement the currently available antibiotics. Last but not least, a simple rapid tool that combines a state-of-the-art FTIR technique with whole-genome sequencing was presented for the diagnosis and identification of infectious bacterial outbreaks. Overall, this editorial summarizes many interesting approaches to combat the global problem of antibiotic resistance, encouraging further research to mitigate the crisis.

## Author contributions

All authors listed have made a substantial, direct, and intellectual contribution to the work and approved it for publication.
